# The efficacy of vancomycin-loaded biphasic calcium phosphate bone substitute in the promotion of new bone growth and the prevention of postoperative infection

**DOI:** 10.3389/fbioe.2022.988436

**Published:** 2022-11-01

**Authors:** Shi-Yong Wang, Ru-Bin Yao, Kai-Shun Yang, Huang-Chien Liang, Chen-Ying Su, Hsu-Wei Fang, Sher-Wei Lim

**Affiliations:** ^1^ Department of Spinal Surgery, First Affiliated Hospital of Dali University, Dali, China; ^2^ Department of Materials Engineering, Ming Chi University of Technology, New Taipei City, Taiwan; ^3^ Department of Chemical Engineering and Biotechnology, National Taipei University of Technology, Taipei, Taiwan; ^4^ Institute of Biomedical Engineering and Nanomedicine, National Health Research Institutes, Zhunan Township, Taiwan; ^5^ Department of Neurosurgery, Chi-Mei Medical Center, Chiali, Tainan, Taiwan; ^6^ Department of Nursing, Min-Hwei College of Health Care Management, Tainan, Taiwan

**Keywords:** biphasic calcium phosphate, bone substitute, vancomycin, bone formation, osteoporosis

## Abstract

**Background:** Due to the increasing need for suitable alternatives to bone grafts, artificial bones made of biphasic calcium phosphate (BCP) are currently being extensively researched. These porous bone substitutes have also demonstrated considerable incorporation with the host bone, and new bone is able to grow within the porous structure. They therefore offer a potential therapeutic approach for bone defects.

**Methods:** Vancomycin-loaded Bicera™, a BCP bone substitute, was investigated in order to prevent implant-associated osteomyelitis and postoperative infection after orthopedic surgery. The loading capacity of Bicera™ was measured to understand its potential antibiotic adsorption volume. An antibiotic susceptibility test was also carried out to analyze the effect of Bicera™ loaded with different concentrations of vancomycin on the growth inhibition of methicillin-resistant *Staphylococcus aureus* (MRSA). Vancomycin-loaded Bicera™ was implanted into rabbits with bone defects, and general gross, radiographic, and histological evaluation was undertaken at 4, 12, and 24 weeks after implantation.

**Results:** The maximum loading capacity of vancomycin-loaded Bicera™ was 0.9 ml of liquid regardless of the vancomycin concentration. Antibiotic susceptibility tests showed that vancomycin-loaded Bicera™ inhibited the growth of MRSA for 6 weeks. In addition, animal studies revealed that new bone grew into the vancomycin-loaded Bicera™. The percentage of new bone formation from 4 to 24 weeks after implantation increased from 17% to 36%.

**Conclusion:** Vancomycin-loaded Bicera™ could effectively inhibit the growth of MRSA *in vitro*. It was found to incorporate into the host bone well, and new bone was able to grow within the bone substitute. The results of this study indicate that vancomycin-loaded Bicera™ is a potential bone substitute that can prevent implant-associated osteomyelitis and postoperative infection.

## Introduction

Implant-associated osteomyelitis and postoperative infection are often devastating complications for patients who undergo orthopedic surgery (Hernández-Vaquero et al., 2013; Inzana et al., 2016). These complications may lead to re-implantation and cause a higher number of hospital admissions and increased expenses (Badia et al., 2017). Current treatments include surgical debridement and long-term antibiotic administration (Bue et al., 2018). However, the typical antibiotic treatment—which is administered orally or *via* intravenous injection—might not be effective because of poor penetration or poor blood supply at the infection site (Luo et al., 2016). Therefore, using antibiotic-loaded bone substitutes offers an effective method for the prevention of postoperative infection.

The most common pathogen in orthopedic infections is *Staphylococcus aureus* (*S. aureus*), which typically causes early and late infection according to the onset of symptoms after surgery (Gbejuade et al., 2015). Of all the different types of *S. aureus*, methicillin-resistant *S. aureus* (MRSA) infection presents a unique therapeutic challenge (Bue et al., 2018). Currently, vancomycin is used as an effective antibiotic for treating infections caused by MRSA (Li et al., 2010).

Antibiotic-loaded polymethyl-methacrylate (PMMA) beads are used to treat orthopedic infections, as they release a high dose of antibiotics locally at the site of infection (Luo et al., 2016). However, this treatment has some disadvantages: first, the temperature can increase to 100°C during the polymerization process, resulting in a limitation to the incorporation of heat-sensitive antibiotics; second, PMMA beads are not biodegradable, so they need to be removed later (Luo et al., 2016); and last, although antibiotic-loaded PMMA beads release a high dose of antibiotics initially, they do not ensure a prolonged effect of antibiotic treatment (Anagnostakos et al., 2009; Balato et al., 2015). For these reasons, bioceramics have become a suitable alternative.

Bioceramics can be divided into two types according to their biological performance: bioinert ceramics and bioactive ceramics. Bioinert ceramics, such as silicon carbides, alumina, zirconia, and pyrolytic carbon, are commonly used for load-bearing implants (Diaz-Rodriguez et al., 2018), while bioactive ceramics, such as calcium phosphates, bioactive glasses, and aluminum oxides, have been developed to integrate into the body (Diaz-Rodriguez et al., 2018). Calcium phosphates in particular are used for bone repair because of their similarities to bone composition (Gao et al., 2014). There are different types of calcium phosphates, including hydroxyapatite (HAp) and two crystalline forms of tricalcium phosphate (α-TCP and β-TCP). Although the chemical composition of HAp is closer to that of bone, the low solubility and slow degradation rate restricts its use (Zhou and Lee, 2011; Fernandez de Grado et al., 2018). Furthermore, TCP has been shown to be osteoconductive, but the rapid degradation rate results in bad mechanical support during the required time (Cunha et al., 2013). Therefore, a composite ceramic using both HAp and TCP, called biphasic calcium phosphate (BCP), has been used to overcome the disadvantages of HAp and TCP (Chen et al., 2017).

A porous BCP bone substitute, Bicera™, has been previously studied and showed good integration with host bone (Chen et al., 2017). However, the efficacy of Bicera™ in bone growth and postoperative infection remains unclear. Therefore, in the present study, we aimed to investigate the loading efficacy of Bicera containing vancomycin. Based on the vancomycin-loaded Bicera^TM^, The release rate of Bicera^TM^ loaded with different concentrations of vancomycin and its inhibitory effects on MRSA were investigated *in vitro*. To examine whether vancomycin inhibits bone tissue growth, we further implanted vancomycin-loaded Bicera^TM^ into rabbits to evaluate its biocompatibility and bone regeneration.

## Methods

### Materials

The bone substitute used in this study was Bicera™ (Wiltrom Co., Ltd., Taiwan), which was constituted of BCP containing 60% HAp and 40% TCP, with a porosity of 77.7 ± 0.6%. Vancomycin hydrochloride (Biovision Inc., United States) was dissolved in sterilized distilled water at a concentration of 50, 100, or 200 mg/ml.

### Liquid adsorption analysis

A total of 1 g Bicera™ was placed on a plate, to which 0.1 ml of Vancomycin solution containing 50, 100, or 200 mg/ml of vancomycin was added and stirred in ten times for about 100 s. The plate was then tilted to check the leakage of liquid. Equal amounts of water were recorded and added until the leakage could hold the largest loading volume. This method was tested three times.

### Antibiotic susceptibility test

The MRSA from the American Type Culture Collection (ATCC 33593) was grown on tryptic soy agar (TSA, Bacto™, BD, United States) for 12–16 h. The colony was picked and further expanded in tryptic soy broth (TSB, BD Bacto™, BD, United States). Following incubation, the bacterial cell suspension was adjusted to match the turbidity of a 0.5 McFarland standard (getattachment, 2594). The adjusted cell suspension was then spread gently on a TSA plate using sterile swabs. Disks with 5 mg of vancomycin (control), Bicera™ only, or Bicera™ loaded with 50, 100, or 200 mg/ml vancomycin were then placed on plates, the plates were incubated at 37°C for 18 h, and the zone sizes were measured using a ruler. These zone diameters showed sensitivity to MRSA. All samples were sub-cultured to a new agar plate every week, and the diameter of the zone size was measured for a further 5 weeks.

### Animal study

This study was carried out in compliance with the Animal Research: Reporting of *In Vivo* Experiments guidelines. Eighteen female New Zealand rabbits (4 months old, body weight 2.2–3.5 kg, Resource: BioLASCO Taiwan Co., Ltd., Taiwan) were used, and the procedure was approved by the Institutional Animal Care and Use Committee (IACUC) of Master Laboratory Co., Ltd. (IACUC approval number: MS20160701). The rabbits were anesthetized by intramuscular injection with a mixture of 0.5 ml/kg Zoletil (Virbac, Franch) and 0.5 ml/kg Rompun (BAYER KOREA LTD., KOREA), and local skin pain was prevented by subcutaneous injection of 2% Xylocaine (Recipharm Monts, France). The lateral femur condyles of both thighs were exposed after a 2.5-cm skin incision was made. A bone defect 5 mm in diameter and 10 mm in depth was then created using an electric drill. The defect was flushed with saline to remove the bone debris, after which it was filled with bone substitute. The muscle tissue and surface skin were sutured after implantation. Incisions were covered with iodine ointment and wrapped with gauze to prevent inflammation or infection. Bicera™ without vancomycin (the BC group) and Bicera™ with 100 mg/ml of vancomycin (the BV group) were both implanted into 12 femurs, while another 12 femurs were left cavernous as the empty control (the control group).

The femoral defects were evaluated at 4, 12, and 24 weeks after surgery, and all animals were humanely euthanized at 4, 12, and 24 weeks according to the guidelines set forth by the American Veterinary Medical Association Panel on Euthanasia (JAVMA, March 2000). Body weights were measured and recorded immediately before euthanasia. The left and right femora of the rabbits were then collected, and tissue samples were placed in 10% formalin. Necropsy observations were also recorded.

All femur samples were harvested at the designated time points and radiographed in the anteroposterior and lateral planes to evaluate the contact surface between bone substitute and bone tissue. The femoral condyles were subsequently fixed in 10% formalin for 24 h, followed by decalcification using a hydrochloric acid decalcifier solution for 14 days. For histological analysis, samples were embedded in paraffin wax and sectioned along the long axis of the femur. These sections were mounted on glass slides and stained with hematoxylin and eosin. The assessment included the presence of inflammatory cells between the femoral tissue and implanted article, the interface between the bone tissue and implanted article, and the amount of bone ingrowth. These sections were also collected for histomorphometric analysis. The sections were calculated using an image analysis technique (Image-Pro Plus, Media Cybernetics, Inc., Maryland, United States) that recognizes newly formed bone, lipid cells, bone marrow cells and implants based on different red, green, and blue values from a digitalized image. The percentage of new bone formation was then determined as the ratio of the new bone formation area to the cross-sectional area.

### Statistical analysis

All data among different groups were compared using one-way analysis of variance with Turkey’s Honest Significant Difference test. A value of *p* < 0.05 was considered statistically significant.

## Results

### The capacity of vancomycin volume for Bicera™

In order to confirm the largest drug capacity of Bicera™, three different concentrations of vancomycin were loaded onto 1 g of Bicera™. It was found that around 0.9 ml of vancomycin could be loaded onto 1 g of Bicera™ regardless of the concentration of vancomycin (see Table 1). The absorption method of different vancomycin concentrations was used in Antibiotic susceptibility tests and Animal studies.

**TABLE 1:** The largest loading volume of vancomycin onto 1 G of Bicera^™^.

Concentration of vancomycin (mg/ml)	Group 1 (ml)	Group 2 (ml)	Group 3 (ml)	Average (ml)
50	0.93	0.87	0.88	0.89
100	0.91	0.90	0.92	0.91
200	0.88	0.89	0.94	0.90

### The efficacy of vancomycin-loaded Bicera™ in the inhibition of bacteria growth

The efficacy of vancomycin-loaded Bicera™ in the inhibition of bacteria growth was evaluated. After vancomycin-loaded Bicera™ was placed on an MRSA plate for a week, the zone size was observed (see Table 2). The results showed that, when Bicera™ contained a higher concentration of vancomycin, the zone size was larger. To understand whether vancomycin-loaded Bicera™ could maintain long-term efficacy, samples were moved to a new MRSA plate for the subsequent 5 weeks. Although the zone size was decreased at week 6 (see Table 2), the diameters of the inhibition zones ranged from 19.6 ± 0.9 to 10.9 ± 0.7 mm, suggesting that vancomycin could be continuously released for 6 weeks.

**TABLE 2:** The inhibition zone size of MRSA caused by vancomycin alone or vancomycin-loaded Bicera^™^.

	Week 1 (mm)	Week 2 (mm)	Week 3 (mm)	Week 4 (mm)	Week 5 (mm)	Week 6 (mm)
Vancomycin alone	18.4 ± 0.4	18.8 ± 1.6	18.1 ± 0.2	18.3 ± 0.6	18.2 ± 0.4	17.6 ± 0.5
Bicera™ + 50 mg/ml vancomycin	18.9 ± 0.4	13.9 ± 1.3	10.9 ± 0.4	11.7 ± 1.1	11.0 ± 0.8	9.9 ± 0.3
Bicera™ + 100 mg/ml vancomycin	19.6 ± 0.9	15.6 ± 1.7	14.1 ± 1.7	13.4 ± 1.0	12.2 ± 0.6	10.9 ± 0.7
Bicera™ + 200 mg/ml vancomycin	21.0 ± 1.2	16.5 ± 1.4	14.2 ± 1.2	14.6 ± 0.2	13.8 ± 0.7	13.1 ± 0.7

### The efficacy of Bicera™ alone and vancomycin-loaded Bicera™ in the promotion of new bone formation

**FIGURE 1:**
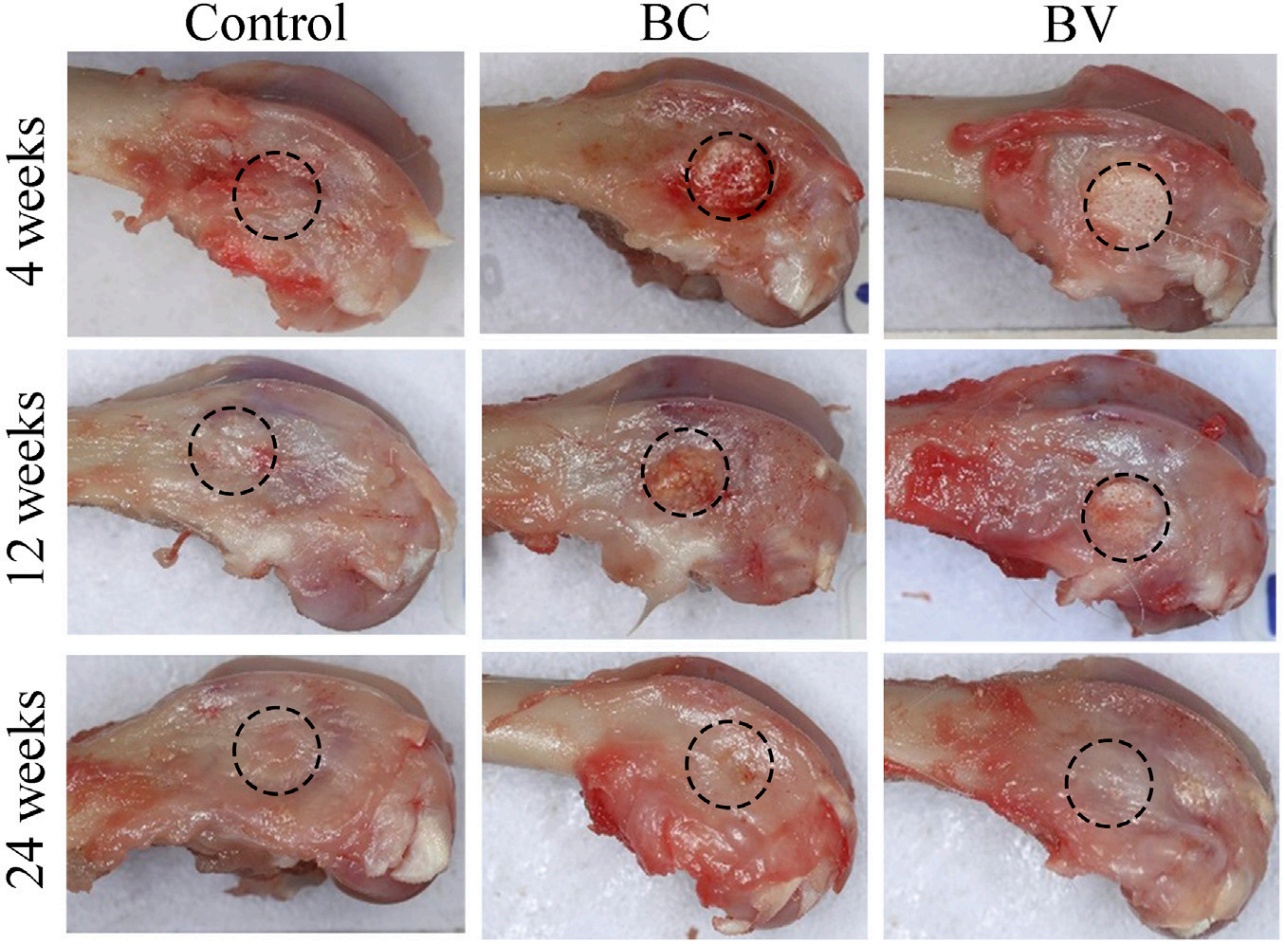
General gross observation of rabbit femur condyle. The implantation site was healed, and no abnormalities were observed in the control, BC, or BV femoral condyles at 4, 12, and 24 weeks after implantation. The black dashed circle shows the implant site. (BC, Bicera™; BV, Vancomycin-loaded Bicera™.)

The general gross observation of the femur condyles showed no obvious abnormality at the defect sites in any of the three groups. At 4 and 12 weeks after implantation, residual Bicera™ with or without vancomycin could be observed (see Figure 1), but at 24 weeks after implantation, Bicera™ with or without vancomycin was not observed.

**FIGURE 2:**
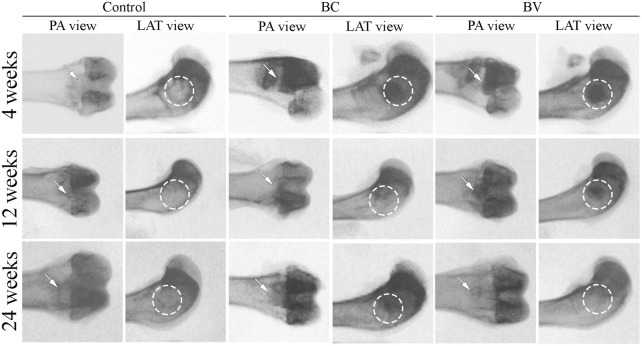
Radiographic images of rabbit femur condyle. The defect without implantation control remained an empty cavity. BC and BV were clearly observed at 4 weeks post-implantation, but the boundary between the implant and the surrounding bone started to become indistinct after 12 weeks. At 24 weeks post-implantation, the boundary was indistinguishable. The white arrow and white dashed circle represented the implant sites. PA, Post-anterior view. LAT, Lateral view. (BC, Bicera™; BV, Vancomycin-loaded Bicera™.)

The radiographic images of the femur condyles after 4 weeks of implantation in the BC and BV groups clearly showed the formation of cylinders in the defect site of the distal femur in the posteroanterior and lateral views (see Figure 2). At 12 weeks post-implantation, the boundaries between the implant and the surrounding bone became indistinct in the BC and BV groups, and at 24 weeks post-implantation, the boundaries between the implant and the surrounding bone merged and became indistinguishable in these groups. In the control group, however, a hole remained even after 24 weeks. The results of the radiographic observation suggest that the degradation of Bicera™ was obvious at 24 weeks post-implantation.

**FIGURE 3:**
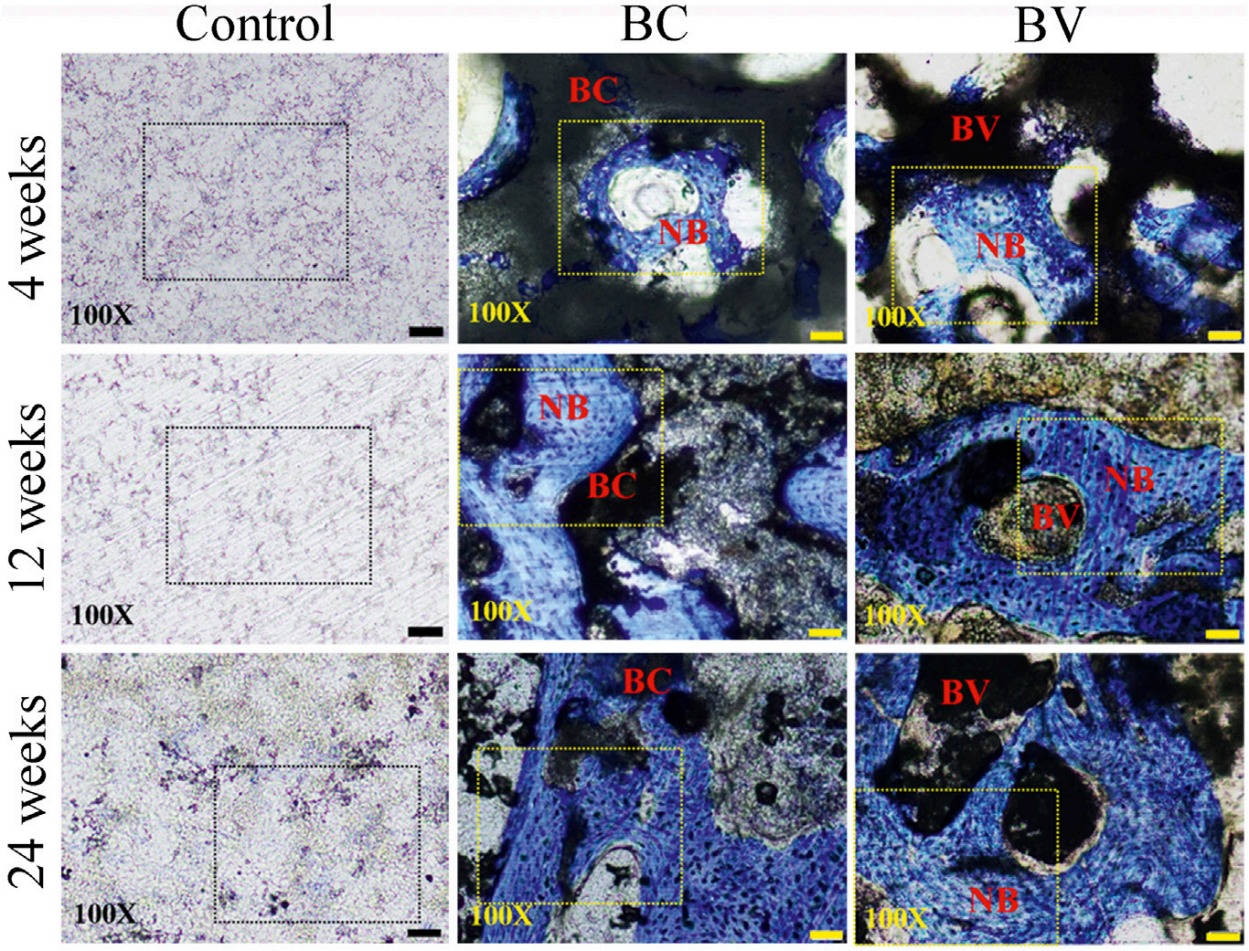
Histological image of rabbit femoral condyle tissue section. In the control group, no fibrous tissue or inflammation response could be observed around the defect site (black dashed rectangle). In the BC and BV groups, new bone cells (blue area inside the yellow dashed rectangle) could be observed at 4, 12, and 24 weeks after implantation. The scale bar represents 50 μm in 100X. NB, New bone. (BC, Bicera™; BV, Vancomycin-loaded Bicera™.)

The histological results showed that there was no detectable inflammation or abnormality at the implant sites (see Figure 3). At 4 weeks after implantation, the residual Bicera™ and vancomycin-loaded Bicera™ were intact, and new bone tissue had grown into the porous structure of the material. The tissue and material had directly adhered together without obvious fibrous tissue or soft tissue, showing good biocompatibility. At 12 weeks after implantation, only parts of the residual material remained intact and with a porous structure. Some of the residual material started to degrade, with the amount of remaining implant being 32.94% and 29.09% in the BC and BV groups, respectively (see Table 3). The amount of remaining implant was only 23% at 24 weeks post-implantation for both the BC and BV groups, and new bone formation was more obvious, although there was no statistically significant difference between the two groups (*p* = 0.1118) (see Figure 3 and Table 3). The area of new bone growth increased from 21.70% to 37.11% in the BC group and from 17.66% to 36.44% in the BV group, suggesting that adding vancomycin does not affect the osteogenesis ability of Bicera™ (see Table 3). In addition, there was no statistically significant difference in the amount of remaining implant in the BC and BV groups (*p* = 0.8564), indicating that the degradation rate of vancomycin-loaded Bicera™ was similar to that of Bicera™ alone.

**TABLE 3:** Summary of histomorphometric analysis.

Weeks post-implantation	Treatment	Percentage	Remaining	Void space area (%)
		Bone area (%)	Implant (%)	
4 weeks	BC	21.70 ± 7.44	51.73 ± 8.22	26.57 ± 6.92
	BV	17.66 ± 7.48	58.36 ± 7.77	23.98 ± 7.77
	Control	2.00 ± 0.92	N/A	98.00 ± 0.92
12 weeks	BC	31.32 ± 5.91	32.94 ± 6.96	35.41 ± 10.72
	BV	28.08 ± 4.71	29.09 ± 10.17	42.83 ± 12.37
	Control	1.95 ± 0.92	N/A	98.05 ± 0.92
24 weeks	BC	37.11 ± 10.65	23.02 ± 7.22	39.87 ± 11.14
	BV	36.44 ± 7.60	23.26 ± 8.12	40.30 ± 8.17
	Control	1.90 ± 0.76	N/A	98.10 ± 0.76

BC: Bicera™; BV: Vancomycin-loaded Bicera™; N/A: Not applicable.

## Discussion

The method of loading molecules into bone substitute scaffolds can be conducted by adsorption, physical entrapment, and covalent bonding (Diaz-Rodriguez et al., 2018). In this study, adsorption was used for the loading of Bicera™ with vancomycin, and it has been shown that the porous structure of Bicera™ plays a role in the adsorption process (Parent et al., 2017). Bicera™ is a highly porous scaffold with a porosity of 78% and a pore size of 462 μm. The result of the loading capacity test showed that Bicera™ can absorb 0.9 ml of vancomycin solution, and the concentrations of vancomycin do not affect its loading capacity. Having a large loading capacity suggests that Bicera™ can be loaded with different concentrations of vancomycin or combinations of different antibiotics, so the possibilities for treating osteomyelitis clinically can be investigated in the future.

Although Bicera™ can be loaded with 0.9 ml of vancomycin solution, its antibiotic release profile is more important for the treatment of osteomyelitis. The challenge of antibiotic-loaded calcium phosphate bone substitutes is controlling the release of an appropriate dose for a prolonged duration. It has been shown that HAp alone has an initial burst of antibiotic release, and β-TCP alone has a short duration of release (Kundu et al., 2010). In contrast, Bicera™ is composed of a combination of HAp and β-TCP, and the result of this study’s antibiotic susceptibility test demonstrated that vancomycin-loaded Bicera™ can inhibit the growth of MRSA for at least 6 weeks (see Table 2). Therefore, vancomycin-loaded Bicera™ is a better antibiotic carrier than HAp or β-TCP alone.

The porous structure of bone substitutes not only affects the adsorption of antibiotics but also influences scaffold resorption and tissue ingrowth (Mate-Sanchez de Val et al., 2014; Ginebra et al., 2018). Bicera™ has been previously shown to promote new bone formation in rabbits at 12 weeks post-implantation, and the Bicera™ bone substitute was still intact (Chen et al., 2017). When using Bicera™ to treat patients with bone fractures, bone healing occurred in 66.67% of patients within 6 months, suggesting that Bicera™ can promote new bone growth *in vivo* (Chen et al., 2017). Triosite™, another BCP bone substitute, has the same composition as Bicera™ but with lower porosity (70%). New bone formation has been observed when implanting Triosite™ into rabbits, with the percentage of new bone formation being 12%, which is similar to that of Bicera™ implantation (14%) (Chen et al., 2017). In the present study, the percentage of new bone growth was even higher (37%) at 24 weeks post-implantation, confirming that Bicera™ promotes new bone formation. The remaining Bicera™ implant was decreased to 23% after 24 weeks, suggesting that Bicera™ can continuously promote bone tissue growth after 6 months.

The present study found that, after loading with vancomycin, the effect of Bicera™ on new bone formation was not affected. The percentage of bone formation was 36% at 24 weeks post-implantation when implanting with vancomycin-loaded Bicera™, and this percentage was similar to that of implantation with Bicera™ alone. Similar results were observed when implanting a vancomycin-loaded calcium phosphate-calcium sulfate composite in rats with infection, in which new bone formation as well as the inhibition of bacterial growth occurred (Boyle et al., 2019). In addition, vancomycin-loaded HAp has been shown to effectively treat chronic osteomyelitis in a rabbit model after treatment for three or 4 weeks (Shirtliff et al., 2002; Joosten et al., 2004; Peng et al., 2021). Therefore, whether vancomycin-loaded Bicera™ can treat or even prevent infection *in vivo* requires further investigation. In a previous *in vitro* experiment, 1 g of vancomycin was dissolved in 200 ml saline to simulate clinical vancomycin use process, and osteoblasts were immersed in it for 20 min. The results showed that the proliferation of osteoblasts was decreased by 3.5 mmol/L vancomycin by 15%–20%, which returned to the control level within 72 h (Philp et al., 2017). Therefore, it was unknown whether the continuous release of vancomycin-loaded BiceraTM (6 weeks) would cause persistent adverse effects on osteoblasts. The *in vivo* experiments found that sustained-release vancomycin did not affect osteogenesis at 4, 12, and 24 weeks, which may also be related to the osteogenic properties of BiceraTM and the decrease in the amount of sustained-release vancomycin. Further experimental verification is needed.

There are some limitations in the present study. Firstly, we only observed the inhibitory effects of vancomycin-loaded BiceraTM on MRSA but did not perform an *in vitro* quantification of the release profile of the antibiotic from the vancomycin-loaded BiceraTM at different time points. Therefore, it was uncertain whether the release amount had reached the therapeutic dose after 6 weeks. Secondly, vancomycin-loaded BiceraTM should be made into 50, 100, 200, 300, 400 mg/ml solution to better explore the its inhibitory effects on bone growth. The current data only showed that vancomycin-loaded BiceraTM at a concentration of 200 mg/L did not affect bone formation. Thirdly, *in vitro* morphological characterization of Bicera TM alone and vancomycin-loaded BiceraTM by scanning electron microscopy was not performed. Lastly, the observation time of this study was short. In clinical practice, osteomyelitis and postoperative spinal infection often require longer treatment. Further investigations with long follow-up will be needed.

## Conclusion

The present study investigated the effectiveness of vancomycin-loaded Bicera™ in the inhibition of MRSA growth *in vitro* and the promotion of new bone formation *in vivo*. The results showed that vancomycin-loaded Bicera™ can continuously inhibit the growth of MRSA for 6 weeks, regardless of the vancomycin concentration. In a rabbit femoral condyle defect model, new bone formation was observed in the group treated with vancomycin-loaded Bicera™ as well as the group treated with Bicera™ alone without statistically significant differences from 4 to 24 weeks post-implantation. The results suggest that vancomycin-loaded Bicera™ can potentially be used to treat implant-associated osteomyelitis and postoperative infection.

## Data Availability

The original contributions presented in the study are included in the article/supplementary material, further inquiries can be directed to the corresponding authors.
